# Rapid Adaptation of Night Vision

**DOI:** 10.3389/fpsyg.2018.00008

**Published:** 2018-01-23

**Authors:** Adam Reeves, Rebecca Grayhem, Alex D. Hwang

**Affiliations:** ^1^Department of Psychology, Northeastern University, Boston, MA, United States; ^2^John A. Volpe National Transportation Systems Center, Cambridge, MA, United States; ^3^Schepens Eye Research Institute, Massachusetts Eye and Ear, Department of Ophthalmology, Harvard Medical School, Boston, MA, United States

**Keywords:** mesopic vision, scotopic vision, adaptation, vision recovery, HDR

## Abstract

Apart from the well-known loss of color vision and of foveal acuity that characterizes human rod-mediated vision, it has also been thought that night vision is very slow (taking up to 40 min) to adapt to changes in light levels. Even cone-mediated, daylight, vision has been thought to take 2 min to recover from light adaptation. Here, we show that most, though not all adaptation is rapid, taking less than 0.6 s. Thus, monochrome (black-white-gray) images can be presented at mesopic light levels and be visible within a few 10th of a second, even if the overall light level, or level of glare (as with passing headlamps while driving), changes abruptly.

## Introduction

Daylight scenes cover the photopic range (10 to 10^8^ cd/m^2^), in which color is visible, but real-world outdoor night scenes in bright moonlight only span 0.01 to 0.1 cd/m^2^, scenes under outdoor lighting span 0.1 to 1.0 cd/m^2^ (Le Grande, 1957), and lit roads reach only 1 to 2 cd/m^2^ in the carriageway (Ekrias et al., 2008). Thus, most nighttime scenes fit into the mesopic (10^-3^ to 10^0.5^ cd/m^2^) or scotopic (10^-3^ to 10^-6^ cd/m^2^) ranges. The visibility of stimuli presented in steady conditions of illumination has been widely studied and reviewed (Le Grande, 1957), but visibility under transient conditions has tended to concentrate on a few standard conditions and is not so well-studied in general. In this paper, we relate visibility to the properties of the rod and cone photoreceptors and the neural pathways in the retina signaled by them; we do not discuss cortical effects although these can also affect adaptation, though to a lesser extent.

At the lowest levels (scotopic: 10^-6^ to 10^-3^ cd/m^2^), where only rods are active, the eye does not respond to the longer wavelengths of light, yellow, orange, and red being invisible to rods, and color vision fails as the signals from rods are unidimensional. Also, rods are not present in the fovea (where photopigments are most densely packed), and relatively rare in the parafovea (the central 2° of the visual field), so detailed vision is missing. Finally, responses to changes in light level is thought to be sluggish as the rods take 20 to 40 min to fully recover after the offset of a bright light (Hecht et al., 1937; Alpern, 1961; Stabell and Stabell, 2003). It is this latter fact that we wish to addres in this paper.

At mesopic levels, cones begin to be active, although the perception of color is still weak due to the photochromatic interval, in which only shades of gray are registered (Lie, 1963), whereas at photopic levels, color becomes visible. Despite signaling by cones, the response to changes in light level is still thought to be relatively sluggish, as cones take up to 2 min to recover in the dark. Slow recovery occurs not only after bleaching the photopigments by exposure to bright light (Hollins and Alpern, 1973; Mahroo and Lamb, 2004), but also after exposure to the lower light levels, which primarily adapt the retinal neurons fed by the cones. Thus typical time-constants for recovery in the dark after moderate light adaptation are of the order of 15 s in the blue–yellow opponent pathway (Pugh and Mollon, 1979) and 16–20 s in the red–green opponent pathway for the recovery of flicker and detection thresholds, respectively (data re-analyzed from Reeves, 1983). Clearly, to the extent that the viewer’s light and dark adaptation lag behind the change in light level, he or she will be able to see fewer scene details than when properly adapted, even if residual sensitivity is sufficient to identify high contrast stimuli.

In favor of mesopic and scotopic vision, however, is the important point that the signals from rods piggy-back via retinal A2 amacrine cells onto the retinal ganglion cells, which integrate the luminance information from long and middle-wavelength sensitive cones with the luminance information from the rods, and whose axons form part of the optic nerve. Thus, although color and foveal details are largely excluded in night vision, the visual brain receives the similar retinal image structure at night as during day. Hence, objects perceived at night have the same spatial dimensions as those seen during the day, and one can walk or run unscathed through fairly dense woods with nothing more to guide one than moonlight (<0.1 cd/m^2^). Indeed, rods provide useful visual signals up to 6 cd/m^2^ (Aguilar and Stiles, 1954) and dominate peripheral vision over much of the mesopic range, as shown by the B-wave of the electro-retinogram and the visual evoked potential (Korth and Armington, 1976). They also affect the operation of the cones (Goldberg et al., 1983; Frumkes and Eysteinsson, 1988; Stabell and Stabell, 2003; Zele et al., 2008). The fact that rods may provide useful visual information even at low luminance levels has never been denied, and (hardly surprisingly) is explained by the retinal physiology.

If the standard picture of sluggish dark adaptation was always true, then visual stimuli would be invisible until the viewer’s vision regained sufficient sensitivity to see scene details or motion. Perception of a moonlit scene would be impossible for minutes after a glare source has passed by. The classic studies of dark adaptation indeed demonstrate that slow recovery is typical after exposure to bright light, but a few studies have indicated that recovery from dimmer lights is much faster. For example, Baker (1961) reported that rods recovered fully within 0.6 s of extinguishing a steadily illuminated background field of 0.03 cd/m^2^. In the case of a brief glare source similar to oncoming headlights, recovery takes 0.8 s for younger subjects (Van Derlofske et al., 2005), although longer (2.1 s) for older subjects tested with low-contrast targets (Schieber, 1994). Therefore the standard course of dark adaptation as portrayed in numerous texts may be somewhat misleading. Indeed, although the dark adaptation curves, as shown in (Alpern, 1961; Makous and Boothe, 1974; and many other studies), have a valuable place in studies of receptor physiology, they rarely apply to natural vision because illumination in nature does not change immediately from bright to black, as in the typical experiment, but changes over an extended period during dawn or dusk.

On the other hand, if artificial lighting such as street lights or oncoming headlights are included in the scene, as while driving at night, the standard dark adaptation curve may apply, given that the luminance of streetlights can exceed over 100 cd/m^2^ at visible distances (Eloholma et al., 2004; Ekrias et al., 2008) and the luminance of oncoming headlights can reach more than 10,000 cd/m^2^ as the oncoming car is passing by Hwang and Peli (2013). In the latter case, the highly dynamic motions of the bright spot-lights stimulate retinal receptors in a relatively short time, which initiate local dark adaptation processes after the light source slips away. Full recovery from such dynamic exposures has not been studied, but it is known that light and dark adaptation are highly localized to the area exposed on the retina, and so the great majority of the visual field will not have been adapted to these high levels, but rather adapted to a small fraction of them due to light scattered across the retina by the optics of the eye.

Scatter profiles are complex, but the amount of light scatter has been estimated by Walraven (1973) to be 0.1% of the glare source at 3° of visual angle away from the retinal image of the glare source, and even less further away (e.g., 0.01% at 7°), indicating that most of the retina is adapted to low levels by glare. Interestingly, the interfering effect of glare on vision, known as ‘disability glare,’ may be even less than that predicted from optical scatter alone, as the glare source also serves as a faint background, which increases the sensitivity to contrast (Patterson et al., 2015).

In short, recovery data are needed to estimate visual recovery over a full range of luminance levels, not just the high ones used in the standard literature. Rapid recovery of the luminance pathway during dark adaptation following exposure to moderate light levels has been documented by our lab for both cones (Reeves et al., 1998) and rods (Reeves and Grayhem, 2016). Those data were obtained for theoretical reasons, but here we reanalyzed them, combined with newly acquired data, to delineate the general transient characteristics of rapid dark adaptation of human vision.

## Materials and Methods

In those dark adaptation studies (Reeves and Wu, 1997; Reeves and Grayhem, 2016), the recovery of both target detection and perception of flicker in the dark were studied. Maxwellian view optics, which induce uniformly illuminated light by imaging a light source in a 2 mm spot at the center of the pupil (Westheimer, 1966), were used to circumvent the increase in pupil diameter with decreases in light level, and thus ensure that retinal illuminance was precisely controlled in every condition. Background field luminance level in trolands was obtained by photometry for the white (equal-energy) or monochromatic (500 or 530 nm) fields that were employed in the experiments. One troland (td) is a unit of retinal illumination, i.e., actual illumination after passing through the human viewer’s pupil. The relation between trolands and luminance is complicated as pupil size varies non-linearly with light level, but can be simplified satisfactorily to log-linear regressions for a wide range of luminance levels, namely, L = 10^-6^ to 10^3^ cd/m^2^. To transform trolands back to scene luminances for practical applications, given that the troland level (in td) is the effective pupil area (in mm^2^) multiplied by the luminance L (in nits or cd/m^2^), we only need to know the pupil diameter. For an average young person, the pupil diameter (d) is approximately *d* = 5 - 3 × tanh(0.4 × log(L)) (Le Grande, 1957, p. 96). For example, 1 scotopic td is equivalent to 0.03 cd/m^2^ for a standard observer with the 6.43 mm pupil diameter typical for viewing at this particular level (Linke et al., 2012). For scotopic trolands, regressions indicate that 99.97% of the variance in Le Grande’s table is accounted for by the linear equations,

log⁡(td)=0.9639×log⁡(L)+1.5123, and inversely, log⁡(L)=1.0372×log⁡(td)−1.5696

For photopic vision, the effective troland for white light, assuming standard pupil entry, average pupil diameter, and the Stiles–Crawford effect (as explained by Le Grande, 1957, p. 104 and Westheimer, 2008), is given (with over 98% of the variance accounted for) by the linear equations,

log⁡(td)=0.8526×log⁡(L)+1.1839, and inversely, log⁡(L)=1.1712×log⁡(td)−1.384

These equations were used to convert trolands in the source publications to cd/m^2^ for the current article, to conform usages in the lighting, automotive, and display industries.

**FIGURE 1:**
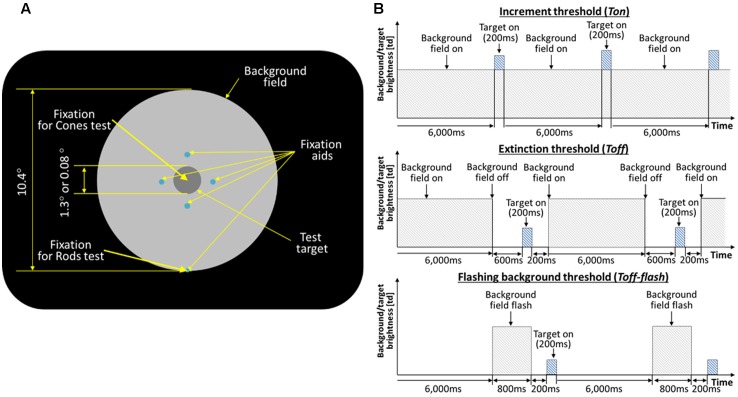
**(A)** Schematic of the stimulus. The large circular patch containing the test disk is a uniform background field of light that defines adapting level. The four small dim spots (0.03°) guide subject’s attention to the test target location. For the rod experiments, the subjects were instructed to fixate at the fixation spot located along the edge of the background disk (5° below the test target). For the cone experiment, the subjects were fixating at the test target. **(B)** Timeline of the test target and background field presentations. The background field appears continuously (*Ton*), disappears temporarily (*Toff*), or flashes (*Toff-flash*). The test target flashed for 200 ms, as shown, or flickered for 2 s (not shown).

For experiments on the recovery of rod vision, thresholds of the test target were obtained while subjects fixated a spot located along the bottom edge of the background field, such that the test target was located at 5° above fixation. For experiments on the cones, thresholds of the test target were measured with subjects fixating at the test target with help of the fixation aids, locating the test target at the fovea. **Figure 1A** shows the schematic view of the stimulus as seen in the Maxwellian view.

On each presentation, observers reported whether the test target was visible or not, or whether it seemed to flicker or not. The test target appeared for 200 ms (**Figure 1B**, top), and subtended either 1.3 or 0.08° of visual angle in different experimental sessions. An adaptive program adjusted the intensity of the test target across trials until it found the minimal level for just seeing 75% of the flashes or flickers. Five such levels were obtained in succession and averaged to give each threshold value, with standard deviations of 0.06 log units or less after practice.

Thresholds were obtained in three conditions: after long-term dark adaptation (the ‘Absolute’ threshold, or *Tabs*), after light adaptation to a steady field (the ‘increment’ threshold, or *Ton*), and just after the adaptation field had been extinguished (the ‘extinction’ threshold, *Toff*; **Figure 1B**, middle). When measuring *Toff*, the background field returned 400 ms after the test target was flashed, and the observer was re-adapted to the background for 6 s between trials to maintain the same level of light adaptation. In the main experiments, the field intensity level was initially very dim, hardly raising the thresholds above *Tabs*, and then was doubled after both *Ton* and *Toff* had been measured. After each doubling, subjects light adapted for 3 min to the new field intensity. The values of experimental interest here, as obtained for each subject and each field level, are the ratios *Ton/Tabs* and *Toff/Tabs*, since the actual values of *Ton* and *Toff* depend on a host of factors such as a subject’s sensitivity and the test wavelength, duration, and eccentricity, not relevant to the issue of recovery and which are compensated for by using the ratios. Test and field parameters were chosen so that thresholds would be mediated by rods in Reeves and Grayhem (2016) and by cones in Reeves et al. (1998). In each study, thresholds were measured for three observers with healthy eyes in each study (none reported vision problems, and all had normal acuity and pupillary responses). Test parameters (size and duration) were fixed for each session, so that *Tabs, Ton*, and *Toff* could be compared directly.

To monitor the course of dark adaptation more closely, *Toff* thresholds were measured in subsidiary experiments at various times from 200 ms to 2 s after turning off the background field (Reeves et al., 1998; Reeves and Grayhem, 2016). In these experiments, just two field intensities were used, which raised *Ton* about 0.5 and about 1.0 log units above *Abs*. Recovery was not exponential, as has been reported after turning off intense fields (Alpern, 1963). Instead, thresholds dropped abruptly after the field was turned off, and then hardly varied over the next second. The ‘elbow’ at which thresholds stopped their abrupt descent and began to recover only very slowly was between 200 and 400 ms in photopic conditions and between 200 and 600 ms in scotopic ones. Therefore, to ensure that all the *Toff* thresholds had reached the elbow, the data reported here from the main experiments have been averaged at 400 ms at photopic levels and at 600 ms at scotopic levels.

Control thresholds (*Ton-flash*) and (*Toff-flash*) were measured with the background appearing for only 800 ms (**Figure 1B**, top and bottom). The eye remained in darkness for 6 s between trials, for both *Ton-flash* and *Toff-flash*. Thus the eye was essentially dark-adapted throughout, except for brief exposures to the field. Otherwise, *Ton-flash* and *Toff-flash* were measured as before, that is, on the background (400 ms after it turned on), and at various times after it was turned off. Since the latter measurements revealed an elbow at 200 ms for every subject at every light level, the *Toff-flash* data were averaged at 200 ms for the present report.

### Ethics Statement

The study protocols were all approved by the institutional review board (IRB) of Northeastern University, and the studies were carried out either by the authors acting as subjects or with the written informed consent from naive subjects.

## Results

**FIGURE 2:**
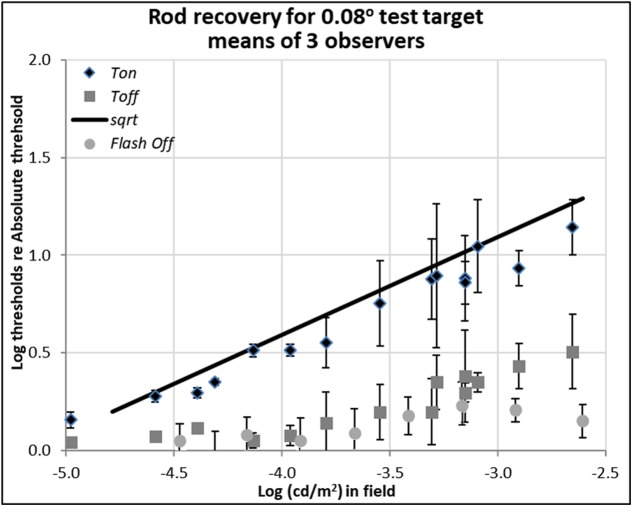
Plots of rod thresholds for detecting a small target (0.08°), either on a steady field (*Ton*) or 600 ms (*Toff*) or 200 ms (*Toff-flash*) in darkness, relative to Tabs. Thresholds were averaged over three observers and are plotted as a function of log (cd/m^2^) in the background field. Bars denote ± 1 standard error of the mean. Solid black line indicates the square-root prediction.

**FIGURE 3:**
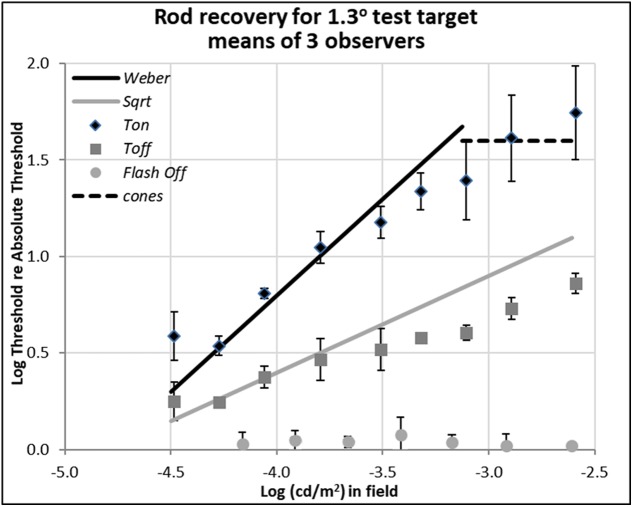
Plots of rod thresholds for detecting a larger target (1.3°), either on a steady field (*Ton*) or 600 ms (*Toff*) or 200 ms (*Toff-flash*) in darkness. Thresholds were averaged over three observers and are plotted as a function of log(cd/m^2^) in the background field. Bars denote ± 1 SE (standard error of the mean) except for *Flash Off*, where only +1 SE is shown. Solid black line indicates the Weber prediction and the gray line indicates the square root prediction.

**FIGURE 4:**
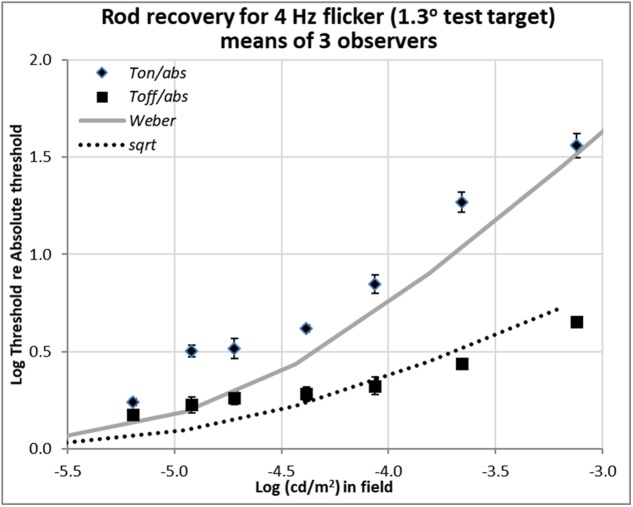
Plots of relative rod thresholds, *Ton/Tabs* and *Toff/Tabs*, for perceiving 4 Hz flicker in a larger test target (1.3°). Thresholds were averaged over three observers and are plotted as a function of log(cd/m^2^) in the background field.

**FIGURE 5:**
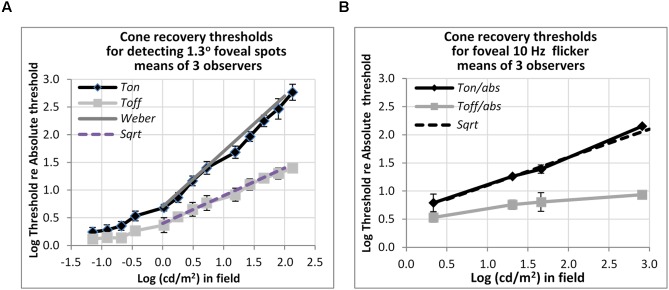
Plots of cone thresholds for **(A)** detecting a larger (1.3°) non-flickering target and **(B)** flickering target (10 Hz). Thresholds were averaged over three observers, and plotted as a function of cd/m^2^ in the background field. Continuous and dotted straight lines show the Weber and square-root predictions for *Ton* and *Toff*, respectively. Note that ‘*abs*’ here refers to the absolute threshold of the cones, not that of the rods, and corresponds (for the 1.3° test) to the dotted line shown in **Figure 3**.

We show the rod data in **Figures 2**–**4** and cone data in **Figure 5**. Data are plotted as log_10_ relative thresholds, *Ton/Tabs* and *Toff/Tabs*, versus log_10_ field intensity levels in cd/m^2^ derived from the original measurements in trolands using Eqs 1 and 2. Thresholds are plotted relative to *Tabs* because the lightest spot intensity needed to reach absolute threshold depends on a host of test factors, such as wavelength composition, duration, and eccentricity, none of which varied between the target ‘on’ and ‘off’ conditions, and which are not germane to the issue at hand, namely, recovery in the dark. In other words, the data points in those figures represent the amount of logarithmic increase of the tested threshold (e.g., *Ton*, *Toff*, and *Toff-flash*) above the corresponding *Tabs* (vertical axis), for given background field level (horizontal axis). Given the use of logarithmic co-ordinates, on very, very dim backgrounds, when the increment threshold (*Ton*) is still equal to the absolute threshold (*Tab)*, the relative threshold, *Ton*/*Tabs* = 1, and so the log_10_ (*Ton*/*Tabs*) value plotted on the y-axis equals zero. On brighter fields, *Ton* > *Tabs* and the relative threshold increases upward by the amount equal to the reduction in log sensitivity. Relative thresholds also remove one source of individual difference, namely, overall sensitivity to the test spot. However, individual differences remain in the sensitivity to the effect of the background light, which shifts the log-log plots laterally, and these are removed when data are averaged across subjects.

Data for the rods was averaged over the three observers only after applying three-point smoothing to each subject’s data, as the rod thresholds obtained at 5° eccentricity are rather variable, unlike the foveal thresholds used to obtain cone data. Smoothing was applied by averaging each set of 3 successive thresholds obtained at 0.3 log unit increments in field intensity. Between-subject variation was about 0.1 log unit for all background conditions, as indicated by standard error bars in the figures. Each observer’s relative threshold data were graphed individually in the earlier reports (Reeves et al., 1998; Reeves and Grayhem, 2016), to which the reader interested in individual differences and the raw data may refer.

In both **Figures 2**, **3**, the data plotted with diamonds and labeled ‘*Ton*’ are so-called ‘increment thresholds,’ that is, the thresholds for just seeing small, brief, probe flashes presented on large, steady, background fields (Stiles, 1939). Therefore, the data for the *Ton* condition represents visual sensitivity after light adaptation. The data plotted with gray squares and labeled ‘*Toff*’ are the ‘extinction thresholds,’ where they were collected just after the offset of the background field, i.e., after plunging the eye into total darkness. Therefore, the data for the *Toff* condition represents visual sensitivity in early dark adaptation. The data points plotted with circles and labeled ‘*Toff-flash’* are for the control condition, where the background field itself was flashed for 800 ms and then the test target showed up 200 ms after the background field was turned off.

### Detection Thresholds of Rods for Small (Spot) Test Target

Rod thresholds for detecting a 0.08° (5′ arcsec) test target under three background field conditions are plotted in **Figure 2**.

The increment thresholds for detection shown by diamonds (*Ton*) lie close to the theoretical Rose-De Vries square-root line, as plotted with a slope of 0.5 in log-log coordinates, in agreement with extensive earlier data (e.g., Barlow, 1957, 1958; Sharpe et al., 1993; Brown and Rudd, 1998).

The extinction thresholds shown by gray squares (*Toff*) show that when steady but dim background field was turned off, thresholds recover almost all the way toward their absolute threshold (about 0.15 log unit above the *Tabs*) within 600 ms. When somewhat brighter background fields were turned off, recovery of sensitivity at 600 ms time point was incomplete, but threshold still remained within a half a log unit of the absolute threshold, as shown by gray squares in **Figure 2**. Note that compared to the 13 log unit span of human vision, standard errors of 0.3 log units or less (shown by bars) are small, but there remain systematic individual differences which contribute to them (see raw data in Reeves and Grayhem, 2016).

The detection thresholds plotted by light gray circles (*Toff-flash*) show thresholds obtained in the dark just 200 ms after the background field had been flashed for 800 ms. The recovery for these conditions is similar to the ones already discussed in *Toff* condition, though slightly faster and more complete: even when relatively brighter background fields were turned off, the corresponding log relative threshold are about 0.25 log unit above the *Tabs* within 200 ms.

### Detection Thresholds of Rods for Larger Test Target

Data shown in **Figure 3** are for rod detection thresholds of larger spots (1.3° in diameter).

In this case, the rod increment threshold (*Ton*) follows the Weber law (marked by the solid black line), being proportional to the field intensity (a slope of 1.0 in log-log scale), rather than its square-root (a slope of 0.5 in log-log scale), as also expected from previous research (Stiles, 1939; Sharpe et al., 1993). The square-root prediction indicated by the solid gray line fits the thresholds measured 600 ms after the field was turned off (*Toff*), marked by gray squares, at least for dimmer backgrounds. At high background levels, increment thresholds are mediated by cones (dotted line, as measured with a 1.3° foveal test flash), and the Weber and square-root predictions for the rods no longer hold.

When comparing the data between *Ton* and *Toff* conditions for the different target sizes, the critical theoretical point is that removing the dim background field removes the photon-driven noise, permitting the extinction thresholds (*Toff*) to fall almost immediately from the Weber law (*Ton*) to the square-root law for 1.3° spots (**Figure 3**), or from the square-root law to *Tabs* for 0.08° spots (**Figure 2**). This result is predicted since the variability in the external (photon-driven) noise is proportional to the mean level (Krauskopf and Reeves, 1980), and turning off the field immediately removes this source of external noise. At even dimmer light levels, not shown here, internal (receptoral and neural) noise or ‘dark light’ becomes increasingly relevant and the predicted curves turn a corner as they asymptote toward *Tabs*. That recovery takes 10th of a second rather than no time at all, can be explained by the integration period of the photoreceptors (Reeves and Grayhem, 2016).

The partial recovery of visibility in the first 600 ms (*Toff*) for the 1.3° test target is more indicative of normal viewing than the full recovery found with the small target, as the latter is smaller than most visual features of interest. However, the very fact that recovery follows the square-root law is encouraging because majority of recovery can be achieved in relatively short time. For example, the increment threshold (*Ton*) of the 1.3° target on the 0.003 cd/m^2^ background field (-2.6 on the log scale in **Figure 3**) is 55 times of absolute threshold (*Tabs)*, but threshold recovered to just 7.4 times of absolute threshold in just 600 ms (*Toff*).

The data plotted by the circular gray data points in **Figure 3**, labeled ‘*Toff-flash,’* show thresholds for the 1.3° test target obtained in the dark, just 200 ms after the background field had been flashed for 800 ms. These thresholds recover well, returning to within 0.05 log units of *Tabs*.

### Detection Thresholds of Rods for Larger Flickering Target

Recent unpublished flicker data from an Abstract (Reeves and Grayhem, 2017), shown in **Figure 4**, were obtained with 1.3° test target stimuli of 500 nm, modulated sinusoidally at 4 Hz for 2 s. The background fields were steady, not flashed. Stimulation was again isolated to rods, and the procedure and apparatus were otherwise as described in Reeves and Grayhem (2016). Flicker thresholds were averaged over three observers (two from the previous study and one new) and are plotted as a function of background luminance.

The flicker thresholds obtained on the steady onset background field (*Ton*), plotted with diamonds, show that 4 Hz flicker was visible close to the detection thresholds indicated in **Figure 4** by the solid gray curve. This behavior was expected since 4 Hz is the peak flicker sensitivity of the rods (Huchzermeyer and Kremers, 2017).

### Detection Thresholds of Cones for Larger Flickering Target

Other measurements show that rapid recovery is also typical for cones. **Figure 5** shows foveal detection thresholds relative to *Tabs* averaged over three observers from Reeves et al. (1998) for detection of non-flickering targets (**Figure 5A**), and thresholds for the perception of 10 Hz flicker averaged from three other subjects in Reeves and Wu (1997) (**Figure 5B**). As before, thresholds were obtained on a steady lit background field (*Ton*) and 200 ms after the background field was plunged into darkness (*Toff*).

For detecting the non-flickering test target for cones (**Figure 5A**), *Ton* thresholds follow the Weber rule and *Toff* thresholds follow the square-root rule. The drop of threshold by one half, on a logarithmic basis, implies a fairly sharp recovery. Thus, for example, with a 1.2 log cd/m^2^ (or 100 td) background field, increment thresholds (*Ton*) are about 40 times, or 1.6 log units above, *abs*, whereas thresholds after just 200 ms in the dark (*Toff*) are about six times (0.8 log units above) *abs*. The 10 Hz flicker targets for cones (**Figure 5B**) also showed rapid recovery: to illustrate, after adaptation to a background field of 1.6 log (cd/m^2^), *Ton* is 1.4 log units (25 times) above *abs*, whereas *Toff* is 0.8 log units (6.3 times) above *abs*, for a recovery of 0.6 log units (4 times) in just 200 ms. However, the recovery, though vivid, was incomplete; recovery was only to about 0.8 log units above *abs*, not all the way. The time-course for complete recovery was not studied, but it may well be slow.

## Discussion

The rod increment thresholds (*Ton*) for the small (0.08°) target (**Figure 2**) follow the square root rule (slope = 0.5 on the log scale). This, the so-called Rose-DeVries law, where the detection thresholds for small rod-mediated test spots rise in proportion to the square-root of background field level (De Vries, 1943; Rose, 1948), is expected from the increase in photon-driven noise with field level. This can be referred to the inherent Poisson variability in the photons delivered by the light source, as photon-driven noise in the receptors is distributed as a compound Poisson, in which variability is a fixed proportion of the mean level (Teich et al., 1987). The noise level against which detections are made is the square root of the sum of the variability due to the field intensity and the (uncorrelated) variability due to intrinsic retinal noise, as postulated by Barlow (1957), since above *Tabs*, the target, at threshold, is too weak to contribute to the total noise, the variability of the photons in the test flash being negligible. Thus, the increment thresholds for the small target follow the Rose-DeVries law. Full recovery to *Tabs* is expected for this target on this basis, since turning off the field removes all the photon-driven noise (Krauskopf and Reeves, 1980) and leaves behind only the intrinsic retinal noise that determines the absolute threshold. Recovery was complete only for the dimmest conditions, but even with brighter rod fields, which presumably partially adapted the rod pathway, the *Toff* thresholds recovered to within 0.05 log units of the absolute threshold.

Note that the control thresholds for this condition (*Toff-flash*) showed fast and almost complete recovery even when the background was relatively bright. Presumably, the 800 ms exposure to the flashed field did not provide sufficient time for the rod pathway to adapt. Important thing here is that this condition is representative of night driving in which a small glare source, e.g., a headlamp in the oncoming traffic at far distance with driver’s gaze shift. In this case, the data indicates that the rods can recover their sensitivity quickly and able to see the night time scene as before.

When the test target was large (1.3°) (**Figure 3**), the increment thresholds (*Ton*) followed not the Rose-DeVries law but rather the Weber law. Since the effect of Poisson variation in the field delivered by the light source is identical for both test sizes, the difference between the two laws illustrates an additional light adaptation which existed for the larger test flash. Presumably, this difference arises because the larger test flash recruits many more rods, and therefore a form of neural gain control is necessary to postpone saturation; in the case of the small test flash, many fewer rods are stimulated and no such neural gain control is needed. This logic assumes that different neural pathways mediate detection of different spot sizes, as would occur if the receptive fields which mediate rod detections are matched in size to the test spots and each receptive field gain is controlled by lateral inhibitory signals (Barlow, 1958). The gain control should then depend on spot size, and indeed, for intermediate size spots, log-log slopes range between 0.5 and 1.0 (Sharpe et al., 1993; Stabell and Stabell, 2003).

Since the external source of noise is removed as soon as the field goes off, recovery is very fast, limited only by the so-called ‘critical duration’ or few 10th of a second over which the receptors integrate photons. However, recovery in gain from the light adaptation generated by the steady field is slow, taking up to 2 min, and so the *Toff* thresholds for the large test spot fall from *Ton*, the Weber line, to the square-root line, not all the way to *Tabs*. Support for this suggestion is provided by the control thresholds for this condition (*Toff-flash)*, where the recovery in threshold for detecting the large test was completed within 200 ms after the background was flashed, presumably because the flashed background did not have sufficient time to light adapt the rod pathway mediating detection. Again, this condition is representative of night driving in which a larger glare source, e.g., a headlamp in the oncoming traffic at near distance passing by, toward larger visual eccentricity, in less than a second. Although our data for the extinction thresholds (*Toff*) for larger test targets following offset of steady fields indicate the final stage of recovery from a prolonged light adaptation can be sluggish, which is compatible with the classic literature on dark adaptation, what we emphasize here is that the initial plunge into darkness corresponds to a vastly improved visual sensitivity, a fact which has been overlooked in the literature.

Importantly, rods and cones both recover quickly in the dark, not only for detecting a single 200 ms pulse, but also for detecting smoothly-varying flicker, at 4 Hz for rods and 10 Hz for cones (**Figure 4**). However, flicker is more complicated than this, and thresholds for perceiving faster flicker (e.g., 20 Hz for cones, 12 Hz for rods) may even go up at field offset (Reeves and Wu, 1997; Reeves and Grayhem, 2017), a phenomenon we called ‘*transient lumanopia*.’ Therefore, we do not maintain that all aspects of vision recover equally well from adaptation, just that at moderate, non-saturating, levels, visual targets become detectable within a few 10th of a second in darkness, even though receptors can take many minutes to fully recover after exposure to brighter light.

The recent development of high dynamic range (HDR) electronic displays that span a wide range of luminance levels, e.g., >1,000 cd/m^2^ (nits) peak level and <0.05 cd/m^2^ black level, makes it feasible to employ the lower end of the scale to make night and twilight scenes visible, even while simultaneously displaying high luminance in the scene. Do our results justify the use of the low intensity range of the new HDR displays? Our data shows that human vision is capable of detecting rapid and slight luminance changes (flicker and presumably motion) in low luminance backgrounds, and sensitivity recovers quickly, if incompletely, within few 10th of a second. Current HDR standards theoretically support only 67 pixel levels (of 0.03 cd/m^2^ steps) for the luminance range from 0 to 2 cd/m^2^. HDR displays would need to squeeze in more pixel luminance levels within this range to fully utilize the capability that human vision can afford. One way this could be implemented on current display hardware is by ‘bit-stealing’ (Tyler et al., 1992), whereby the intermediate luminance levels can be generated by independently modifying each of the RGB component values, since shifts in pixel chroma will be largely invisible at these low light levels. Also, since the critical rate for flicker fusion drops off with the logarithm of field intensity (the Ferry-Porter law; Tyler and Hamer, 1990), to around 12 Hz for rods, temporal compression of the video signal will permit many more intensity levels to be generated at low light levels.

Reading remains a problem at night. As rods are excluded from the high-acuity fovea, most print is invisible to them; for example, a letter would needs to be about 72 points on a hand-held display to be visible to the rods. However, rod adaptation is not altered by exposure to long wavelength lights, as rods are insensitive above about 560 nm, so displays meant to be visible at night can incorporate yellow, orange, and red colors of any luminance level without interfering (much) with rod function. It is true that red light seen only by cones halves rod sensitivity (Makous and Boothe, 1974), but this factor is small compared to the 3 log unit range of rod vision. Thus, a simple solution to the dilemma posed by the need to read letters or symbols is to program them at normal size in yellow, orange, or red, at a light level visible to the cones, and add them to the display.

## Conclusion

Although there are extensive data on rod vision in the stationary case, i.e., with steady illumination, surprisingly little is known about rod vision in more natural conditions in which adapting lights (or glare) are transient. We hope that our studies of detection and flicker will stimulate others to measure in greater detail the spatial and temporal properties of the visual system in early dark adaptation, especially acuity, both for theoretical interest and for the practical demands of HDR displays.

## Author Contributions

AR: contributed in data collection, analysis, and writing the manuscript. RG: contributed in data collection and data analysis for this manuscript. AH: contributed in data analysis and writing the manuscript.

## Conflict of Interest Statement

The authors declare that the research was conducted in the absence of any commercial or financial relationships that could be construed as a potential conflict of interest.
